# 94. Implementation of Antibiotic Prescribing Scorecards in the Ambulatory Care Setting

**DOI:** 10.1093/ofid/ofab466.296

**Published:** 2021-12-04

**Authors:** Sara Revolinski, J Njeri Wainaina, Maxx Enzmann, Deanna Olexia, Christopher Sobczak

**Affiliations:** 1 Medical College of Wisconsin, Milwaukee, WI; 2 Froedtert Hospital, Milwaukee, Wisconsin; 3 Froedtert & the Medical College of Wisconsin, Milwaukee, Wisconsin

## Abstract

**Background:**

Up to 56% of antibiotics prescribed in the ambulatory setting in the United States are inappropriately prescribed, with 30% of those determined to be unnecessary. In order to increase transparency and education about antibiotic prescribing in our ambulatory clinics at our institution, we implemented quarterly scorecards demonstrating antibiotic prescribing trends for primary care prescribers.

**Methods:**

This pre-post interventional study analyzed the impact of prescriber scorecards on antibiotic prescribing, with the intervention consisting of real-time education and presentation of baseline data via scorecards. Prescribers were educated on the scorecard project via live meetings in Nov-Dec 2020. In Dec 2020, prescribers were sent individual emails describing their baseline antibiotic prescription rate (defined as number of prescriptions per 100 patient encounters), de-identified comparison data for other prescribers within their individual clinic, and average rate of the top 10% of prescribers with the lowest prescription rates. Baseline data was from prescriptions dated Jan-Mar 2020. The email also explained the project and shared that quarterly scorecards would be distributed in 2021. Baseline data was compared to prescription data from Jan-Mar 2021. Knowing the COVID-19 pandemic resulted in significantly fewer encounters for respiratory infections, data was also analyzed with respiratory diagnoses removed from the dataset.

**Results:**

In the pre-intervention period, 11,769 antibiotics were prescribed during 92,239 encounters for a prescription rate of 12.8 (95%CI: 12.5-13.0). Of 96,449 encounters in the post-intervention period, 7,326 antibiotics were prescribed for a rate of 7.6 (95%CI: 7.4-7.8; p< 0.0001). When respiratory diagnoses were removed, prescription rates were 6.1 (95%CI: 5.9-6.2) in the pre-group, compared to 6.3 (95%CI: 6.1-6.5; p=0.0546). When analyzed by prescriber, significant decreases were seen in prescriptions by physicians (5.8 vs 5.4, p=0.0035) while increases were seen in prescriptions by advanced practice prescribers.

**Conclusion:**

Antibiotic scorecards sent to prescribers may result in reduced antibiotic prescribing, but further research is needed to elucidate the impact of the scorecards in light of the COVID-19 pandemic.

**Disclosures:**

**All Authors**: No reported disclosures

